# Regularized inverse determination of the tension coefficient in a wave equation

**DOI:** 10.1038/s41598-026-44344-4

**Published:** 2026-04-28

**Authors:** Hakkı Güngör

**Affiliations:** Department of Computer Technologies, Ufuk University, Ankara, Turkey

**Keywords:** Variational methods, Optimization, Regularization, Gradient methods, Laplace transformation method, Engineering, Mathematics and computing, Physics

## Abstract

This study addresses the inverse determination of an unknown tension coefficient in a one-dimensional wave equation from final-time observations. Since coefficient reconstruction is inherently ill-posed, a quadratic Tikhonov regularization term incorporating a prior (initial guess) coefficient is employed to stabilize the minimization. Existence and uniqueness of the regularized minimizer are established. The minimizer is computed by a gradient-type method, where the Fréchet derivative of the objective is obtained via an adjoint problem. For numerical realization, a hybrid scheme that combines a Galerkin approximation with a Laplace-transform technique is used. The methodology is illustrated on two benchmark examples together with a systematic sensitivity study with respect to the regularization parameter and the initial guess; a noise-perturbation protocol is also outlined to assess robustness. The presented framework provides a practical and stable approach for inverse coefficient problems governed by wave equations.

## Introduction

Wave equations serve as fundamental models for describing numerous dynamic processes in physics and engineering, with applications ranging from mechanical vibrations and acoustics to electromagnetics and optics. Many parameter-estimation tasks in these settings are formulated as inverse problems, where the goal is to recover coefficients that cannot be directly measured. In particular, identifying a spatially varying tension (or stiffness) coefficient is important because it controls wave speed and propagation; see, e.g.,^1–3^.

The reconstruction of the tension coefficient is ill-posed: small perturbations in the observations can lead to large deviations in the recovered coefficient. Regularization is therefore essential. In this work, we employ a standard quadratic Tikhonov regularization with a prior coefficient, as in the monographs^4,5^, and develop an adjoint-gradient-based minimization strategy coupled with an efficient numerical discretization.

The contributions of this work are fourfold. First, we establish existence and uniqueness for the regularized identification problem. Second, we derive the Fréchet gradient of the objective by an adjoint (conjugate) formulation, enabling efficient gradient descent iterations. Third, we implement a hybrid numerical realization combining Galerkin approximation and Laplace transformation. Finally, we demonstrate the approach through numerical examples and sensitivity analyses with respect to the regularization weight and the prior coefficient. For recent developments on numerical simulation of wave equations (e.g., meshless space–time RBF methods), see^6^.

1 $$\rho \left(\calligra{\rotatebox[origin=c]{4}{x}} \right){\calligra{\rotatebox[origin=c]{4}{u}}}_{tt}-{\left(k \left(\calligra{\rotatebox[origin=c]{4}{x}} \right){\calligra{\rotatebox[origin=c]{4}{u}}}_{\calligra{\rotatebox[origin=c]{4}{x}}}\right)}_{\calligra{\rotatebox[origin=c]{4}{x}}}=f \left(\calligra{\rotatebox[origin=c]{4}{x}} ,t\right), \left(\calligra{\rotatebox[origin=c]{4}{x}} ,t\right)\in\Omega =\left(0,l\right)\times \left(0,T\right)$$

2 $$\calligra{\rotatebox[origin=c]{4}{u}} \left(\calligra{\rotatebox[origin=c]{4}{x}} ,0\right)=\varphi \left(\calligra{\rotatebox[origin=c]{4}{x}} \right), {\calligra{\rotatebox[origin=c]{4}{u}}}_{t} \left(\calligra{\rotatebox[origin=c]{4}{x}} ,0\right)=\psi \left(\calligra{\rotatebox[origin=c]{4}{x}} \right), \calligra{\rotatebox[origin=c]{4}{x}}\in \left(0,l\right)$$

3 $$\calligra{\rotatebox[origin=c]{4}{u}}\left(0,t\right)=0, \calligra{\rotatebox[origin=c]{4}{u}}\left(l,t\right)=0, t\in \left(0,T\right)$$

Here, $\rho \left(\calligra{\rotatebox[origin=c]{4}{x}} \right)\in {L}_{\infty }\left(0,l\right)$ is density function with

$$0<{\rho }_{1}\le \rho \left(\calligra{\rotatebox[origin=c]{4}{x}} \right)\le {\rho }_{2} \; \text{ almost\; everywhere\; on }\; \left(0,l\right),$$

$k \left(\calligra{\rotatebox[origin=c]{4}{x}} \right)\in {L}_{\infty }\left(0,l\right)$ Is rigidity function with

$$0<{k}_{1}\le k \left(\calligra{\rotatebox[origin=c]{4}{x}} \right)\le {k}_{2} \; \text{ almost\; everywhere \;on }\; \left(0,l\right),$$

$f \left(\calligra{\rotatebox[origin=c]{4}{x}} ,t\right)\in {L}_{2}\left(\Omega \right)$ Is external force function, $\varphi \left(\calligra{\rotatebox[origin=c]{4}{x}} \right)\in {W}_{2}^{1}\left(0,l\right)$ is initial status and $\psi \left(\calligra{\rotatebox[origin=c]{4}{x}} \right)\in {L}_{2}\left(0,l\right)$ is initial velocity. So the solution $\calligra{\rotatebox[origin=c]{4}{u}} \left(\calligra{\rotatebox[origin=c]{4}{x}} ,t\right)$ of the problem (1)–(3) represents the location of the $\calligra{\rotatebox[origin=c]{4}{x}}$ point of the object at time $t$.

In order to control the function $k \left(\calligra{\rotatebox[origin=c]{4}{x}} \right)$, we can introduce the cost functional

4 $$J\left(k\right)={\beta }_{1}\underset{0}{\overset{l}{\int }} {\left[\calligra{\rotatebox[origin=c]{4}{u}} \left(\calligra{\rotatebox[origin=c]{4}{x}} ,T;k\right)-{y}_{1} \left(\calligra{\rotatebox[origin=c]{4}{x}} \right)\right]}^{2}dx+{\beta }_{2}\underset{0}{\overset{l}{\int }}{\left[{\calligra{\rotatebox[origin=c]{4}{u}}}_{t} \left(\calligra{\rotatebox[origin=c]{4}{x}} ,T;k\right)-{y}_{2} \left(\calligra{\rotatebox[origin=c]{4}{x}} \right)\right]}^{2}d\calligra{\rotatebox[origin=c]{4}{x}}$$

and consider the minimization problem of

5 $$\underset{k\in K}{\mathrm{min}}J\left(k\right)$$

on the set

6 $$K=\left\{k \left(\calligra{\rotatebox[origin=c]{4}{x}} \right)\in {L}_{2}\left(0,l\right);0<{k}_{1}\le k \left(\calligra{\rotatebox[origin=c]{4}{x}} \right)\le {k}_{2}\text{ for } \mathop \forall \limits^{ \circ } \calligra{\rotatebox[origin=c]{4}{x}}\in \left(0,l\right)\right\}\subset {L}_{\infty }\left(0,l\right).$$

Here, $${y}_{1} \left(\calligra{\rotatebox[origin=c]{4}{x}} \right), { y}_{2} \left(\calligra{\rotatebox[origin=c]{4}{x}} \right)\in {L}_{2}\left(0,l\right)$$ and $${\beta }_{1}, { \beta }_{2}\ge 0$$, $${\beta }_{1}+{\beta }_{2}\ne 0$$.

With weak solution of problem (1)–(3), one means the function $$\calligra{\rotatebox[origin=c]{4}{u}}\in {C}^{1}\;$$$$(\left[0,T\right], \;$$$${L}_{2}\left(0,l\right))\cap C\left[0,T\right],\;$$$${W}_{2}^{1}\left(0,l\right))$$ satisfying the conditions (2) and (3) and the equality of

7 $$\underset{0}{\overset{T}{\int }}\underset{0}{\overset{l}{\int }}\left[-\rho \left(\calligra{\rotatebox[origin=c]{4}{x}} \right){\calligra{\rotatebox[origin=c]{4}{u}}}_{t}{\eta }_{t}+k \left(\calligra{\rotatebox[origin=c]{4}{x}} \right){\calligra{\rotatebox[origin=c]{4}{u}}}_{\calligra{\rotatebox[origin=c]{4}{x}}}{\eta }_{\calligra{\rotatebox[origin=c]{4}{x}}}\right]d\calligra{\rotatebox[origin=c]{4}{x}}dt-\underset{0}{\overset{l}{\int }}\varphi \left(\calligra{\rotatebox[origin=c]{4}{x}} \right)\eta \left(\calligra{\rotatebox[origin=c]{4}{x}} ,0\right)d\calligra{\rotatebox[origin=c]{4}{x}}=\underset{0}{\overset{T}{\int }}\underset{0}{\overset{l}{\int }}f\eta d\calligra{\rotatebox[origin=c]{4}{x}}dt$$

for each $$\eta \in {L}_{2}\left(\Omega \right)$$.

The problem (1)–(3) can be solved using Laplace Method^1^. The following estimation is also valid for each $k\in K$;

8 $$\underset{0\le t\le T}{\mathrm{max}}{\left[{\Vert \calligra{\rotatebox[origin=c]{4}{u}}\Vert }_{{L}_{2}\left(0,l\right)}^{2}+{\Vert {\calligra{\rotatebox[origin=c]{4}{u}}}_{\calligra{\rotatebox[origin=c]{4}{x}}}\Vert }_{{L}_{2}\left(0,l\right)}^{2}+{\Vert {\calligra{\rotatebox[origin=c]{4}{u}}}_{t}\Vert }_{{L}_{2}\left(0,l\right)}^{2}\right]}^{1/2}{\le c}_{0}\left({\Vert f\Vert }_{{L}_{2}\left(\Omega \right)}+{\Vert \varphi \Vert }_{{W}_{2}^{1}\left(0,l\right)}+{\Vert \psi \Vert }_{{L}_{2}\left(0,l\right)}\right)$$

where the constant ${c}_{0}$ is independent from $f, \varphi$ and $\psi$.

In a final time inverse coefficient determination problem related to a wave equation, generally aims to determine an unknown function in the model so that the final-time state $\calligra{\rotatebox[origin=c]{4}{u}} \left(\calligra{\rotatebox[origin=c]{4}{x}} ,T\right)$ and(or) final time velocity ${\calligra{\rotatebox[origin=c]{4}{u}}}_{t} \left(\calligra{\rotatebox[origin=c]{4}{x}} ,T\right)$ better match the observed final-time state ${y}_{1} \left(\calligra{\rotatebox[origin=c]{4}{x}} \right)$ and final time velocity ${y}_{2} \left(\calligra{\rotatebox[origin=c]{4}{x}} \right)$.

Related inverse function determination and optimal control studies for hyperbolic equations can be found, for example, in^7,2^. Further coefficient identification formulations and numerical strategies for wave-type problems are discussed in^4,7,8^.

On the other hand, it is known that minimization problem (5) is ill-posed. To prove this idea, we can give the following example;

### Example

Considering the domain $$\Omega =\left(\mathrm{0,1}\right)\times \left(\mathrm{0,1}\right)$$, let us choose $${\beta }_{1}=1,$$ $${\beta }_{2}=1$$ and take $${y}_{1} \left(\calligra{\rotatebox[origin=c]{4}{x}} \right)=- \;$$$${{\mathrm{sin}}} \pi x, { y}_{2} \left(\calligra{\rotatebox[origin=c]{4}{x}} \right)=0, p \left(\calligra{\rotatebox[origin=c]{4}{x}} \right)=1, f \left(\calligra{\rotatebox[origin=c]{4}{x}} ,t\right)=0, \varphi \left(\calligra{\rotatebox[origin=c]{4}{x}} \right)= {{\mathrm{sin}}} \pi \calligra{\rotatebox[origin=c]{4}{x}}, \psi \left(\calligra{\rotatebox[origin=c]{4}{x}} \right)=0$$.

Consider the minimization problem (5) such as;

$$J\left(k\right)=\underset{0}{\overset{1}{\int }} {\left[\calligra{\rotatebox[origin=c]{4}{u}} \left(\calligra{\rotatebox[origin=c]{4}{x}} ,1;k\right)-\left(- {{\rm{sin}}} \pi \calligra{\rotatebox[origin=c]{4}{x}}\right)\right]}^{2}d\calligra{\rotatebox[origin=c]{4}{x}}+\underset{0}{\overset{1}{\int }}{\left[{\calligra{\rotatebox[origin=c]{4}{u}}}_{t} \left(\calligra{\rotatebox[origin=c]{4}{x}} ,1;k\right)-\left(0\right)\right]}^{2}d\calligra{\rotatebox[origin=c]{4}{x}} \to {\rm{min}}$$

on the set

$$K=\left\{k \left(\calligra{\rotatebox[origin=c]{4}{x}} \right)\in {L}_{2}\left(\rm{0,1}\right);0<1\le k \left(\calligra{\rotatebox[origin=c]{4}{x}} \right)\le 10 \text{ for } \mathop \forall \limits^{ \circ } \calligra{\rotatebox[origin=c]{4}{x}}\in \left(\rm{0,1}\right)\right\}$$

subject to the problem (1)–(3) with

$${\calligra{\rotatebox[origin=c]{4}{u}}}_{tt}-{\left(k \left(\calligra{\rotatebox[origin=c]{4}{x}} \right){\calligra{\rotatebox[origin=c]{4}{u}}}_{\calligra{\rotatebox[origin=c]{4}{x}}}\right)}_{\calligra{\rotatebox[origin=c]{4}{x}}}=0$$

$$\calligra{\rotatebox[origin=c]{4}{u}} \left(\calligra{\rotatebox[origin=c]{4}{x}} ,0\right)= {\rm{sin}} \pi \calligra{\rotatebox[origin=c]{4}{x}}, {\calligra{\rotatebox[origin=c]{4}{u}}}_{t} \left(\calligra{\rotatebox[origin=c]{4}{x}} ,0\right)=0$$

$$\calligra{\rotatebox[origin=c]{4}{u}}\left(0,t\right)=0, \calligra{\rotatebox[origin=c]{4}{u}}\left(1,t\right)=0$$

The function ${\calligra{\rotatebox[origin=c]{4}{u}}}_{1} \left(\calligra{\rotatebox[origin=c]{4}{x}} ,t\right)= {\rm{sin}} \pi \calligra{\rotatebox[origin=c]{4}{x}} {\rm{cos}} \pi t$ is the solution of the problem for ${k}_{1} \left(\calligra{\rotatebox[origin=c]{4}{x}} \right)=1\in K$ and the value of the functional is $J\left({k}_{1}\right)=0.$ On the other hand, the function ${\calligra{\rotatebox[origin=c]{4}{u}}}_{2} \left(\calligra{\rotatebox[origin=c]{4}{x}} ,t\right)= {\rm{sin}} \pi \calligra{\rotatebox[origin=c]{4}{x}} {\rm{cos}} 3\pi t$ is another solution of the problem for ${k}_{2} \left(\calligra{\rotatebox[origin=c]{4}{x}} \right)=9\in K$ and the value of the functional is again $J\left({k}_{2}\right)=0$. Namely, solution of the problem $J\left(k\right)\to \mathrm{min}$ is not unique on the set $K$ and the problem is ill-posed.

To overcome the ill-posedness, a regularization step is introduced. Specifically, we add a quadratic penalty term $\alpha {\Vert k-{k}^{+}\Vert }_{{L}_{2}\left(0,l\right)}^{2}J\left(k\right)$(weighted by the regularization parameter α > 0) to the data-misfit functional, yielding the regularized objective (9).

9 $$\begin{aligned} I\left(k\right) = & \;{\beta }_{1}\underset{0}{\overset{l}{\int }} {\left[\calligra{\rotatebox[origin=c]{4}{u}} \left(\calligra{\rotatebox[origin=c]{4}{x}} ,T;k\right)-{y}_{1} \left(\calligra{\rotatebox[origin=c]{4}{x}} \right)\right]}^{2}d\calligra{\rotatebox[origin=c]{4}{x}}+{\beta }_{2}\underset{0}{\overset{l}{\int }}{\left[{\calligra{\rotatebox[origin=c]{4}{u}}}_{t} \left(\calligra{\rotatebox[origin=c]{4}{x}} ,T;k\right)-{y}_{2} \left(\calligra{\rotatebox[origin=c]{4}{x}} \right)\right]}^{2}d\calligra{\rotatebox[origin=c]{4}{x}} \\ \quad &+\alpha {\int }_{0}^{l}{\left[k-{k}^{+}\right]}^{2}d\calligra{\rotatebox[origin=c]{4}{x}}. \end{aligned}$$

Here, the function ${k}^{+}$ is used as a prior estimate (and also as an initial guess) for the unknown coefficient. The choice of ${k}^{+}$ can influence the convergence behaviour of the iterative minimization and, in nonconvex settings, the attained local minimizer. Detailed discussions on the roles of both the regularization parameter $\alpha$ and the prior ${k}^{+}$ can be found in^4,5^.

Remark on regularization strategy. The regularizer in (9) is the standard quadratic (Tikhonov) penalty. It is widely used because it yields a smooth convex stabilization term and leads to an explicit adjoint-based gradient. This differs from alternative regularization strategies and parameter-choice rules discussed, for example, in the context of meshless boundary knot methods^10^. In the present work, the novelty lies not in proposing a new regularizer, but in deriving a complete adjoint-gradient formulation and combining it with a Galerkin–Laplace numerical realization for the inverse tension problem.

Hence, we consider the problem of

10 $$\underset{k\in K}{\mathrm{min}}I\left(K\right)$$

Although necessary conditions for identification and controllability in wave equations have been investigated in the literature, the numerical influence of the regularization parameter $\alpha$ and the prior/initial guess function is not always discussed systematically in the inverse-tension setting.

Therefore, besides the theoretical analysis, we include numerical sensitivity studies with respect to $\alpha$ and ${k}^{+} \left(\calligra{\rotatebox[origin=c]{4}{x}} \right)$ and outline a robustness test for noisy final-time observations.

Accordingly, after presenting existence/uniqueness results and the minimization algorithm, we focus on numerical treatments and on the sensitivity of the reconstruction with respect to algorithmic and regularization parameters.

## Existence and uniqueness of the solution for regularized problem

We present the following theorem, which establishes the existence and uniqueness of the solution to the regularized problem (10).

### Theorem

*There exists a dense subset* $G$* of the space* ${L}_{2}\left(0,l\right)$ *such that the problem* (10)* with* $\alpha >0$* has a unique* ${k}^{+}$-*minimum norm solution for any* ${k}^{+}\in G$.

### Proof

Firstly, we must show that the functional $J\left(k\right)$ is continuous on the set $K$. To do this, let us give an increment $\Delta k\in {L}_{\infty }\left(0,l\right)$ to the element $k\in K$ such as $k+\Delta k\in K$.

Then, the difference of the functional $J\left(k\right)$ according to this increment is such as

11 $$\begin{aligned} \Delta J\left(k\right)= & \; \underset{0}{\overset{l}{\int }}2{\beta }_{1}\left[\calligra{\rotatebox[origin=c]{4}{u}} \left(\calligra{\rotatebox[origin=c]{4}{x}} ,T;k\right)-{y}_{1} \left(\calligra{\rotatebox[origin=c]{4}{x}} \right)\right]\Delta \calligra{\rotatebox[origin=c]{4}{u}} \left(\calligra{\rotatebox[origin=c]{4}{x}} ,T\right)d\calligra{\rotatebox[origin=c]{4}{x}} \\ \quad &+\underset{0}{\overset{l}{\int }}2{\beta }_{2}\left[{\calligra{\rotatebox[origin=c]{4}{u}}}_{t} \left(\calligra{\rotatebox[origin=c]{4}{x}} ,T;k\right)-{y}_{1} \left(\calligra{\rotatebox[origin=c]{4}{x}} \right)\right]\Delta {\calligra{\rotatebox[origin=c]{4}{u}}}_{t} \left(\calligra{\rotatebox[origin=c]{4}{x}} ,T\right)d\calligra{\rotatebox[origin=c]{4}{x}}. \end{aligned}$$

Secondly, we will evaluate the terms with $\Delta u$ in this difference. Let ${\calligra{\rotatebox[origin=c]{4}{u}}}_{\Delta }=\calligra{\rotatebox[origin=c]{4}{u}} \left(\calligra{\rotatebox[origin=c]{4}{x}} ,t;k+\Delta k\right)$ be the solution of the problem (1)–(3) according to the element $k+\Delta k$. As a result, the function $\Delta \calligra{\rotatebox[origin=c]{4}{u}}={\calligra{\rotatebox[origin=c]{4}{u}}}_{\Delta }-\calligra{\rotatebox[origin=c]{4}{u}}$ solves the following difference problem;

12 $$\rho \left(\calligra{\rotatebox[origin=c]{4}{x}} \right){\Delta \calligra{\rotatebox[origin=c]{4}{u}}}_{tt}-{\left(k \left(\calligra{\rotatebox[origin=c]{4}{x}} \right){\Delta \calligra{\rotatebox[origin=c]{4}{u}}}_{\calligra{\rotatebox[origin=c]{4}{x}}}\right)}_{\calligra{\rotatebox[origin=c]{4}{x}}}-{\left(\Delta k \left(\calligra{\rotatebox[origin=c]{4}{x}} \right){\Delta \calligra{\rotatebox[origin=c]{4}{u}}}_{\calligra{\rotatebox[origin=c]{4}{x}}}\right)}_{\calligra{\rotatebox[origin=c]{4}{x}}}-{\left(\Delta k \left(\calligra{\rotatebox[origin=c]{4}{x}} \right){\calligra{\rotatebox[origin=c]{4}{u}}}_{\calligra{\rotatebox[origin=c]{4}{x}}}\right)}_{\calligra{\rotatebox[origin=c]{4}{x}}}=0$$

13 $$\Delta \calligra{\rotatebox[origin=c]{4}{u}} \left(\calligra{\rotatebox[origin=c]{4}{x}} ,0\right)=0,\Delta {\calligra{\rotatebox[origin=c]{4}{u}}}_{t} \left(\calligra{\rotatebox[origin=c]{4}{x}} ,0\right)=0$$

14 $$\Delta \calligra{\rotatebox[origin=c]{4}{u}}\left(0,t\right)=0,\Delta \calligra{\rotatebox[origin=c]{4}{u}}\left(l,t\right)=0$$

This difference problem has a unique $\Delta \calligra{\rotatebox[origin=c]{4}{u}}\in {C}^{1}\left(\left[0,T\right],{L}_{2}\left(0,l\right)\right)\cap C\left(\left[0,T\right],{W}_{2}^{1}\left(0,l\right)\right)$ solution in the mean of (7) for each $\Delta k\in {L}_{\infty }\left(0,l\right)$. Furthermore this solution satisfies the following inequality for $\forall t\in \left[0,T\right];$

15 $${\Vert \Delta \calligra{\rotatebox[origin=c]{4}{u}}\Vert }_{{W}_{2}^{\mathrm{1,1}}\left(0.l\right)}\le {c}_{1}\left({\Vert \Delta k\Vert }_{{L}_{\infty }\left(0,l\right)}\right)$$

where ${c}_{1}$ is a constant independent from $\Delta k$ .

Since ${y}_{1} \left(\calligra{\rotatebox[origin=c]{4}{x}} \right)$ and ${y}_{2} \left(\calligra{\rotatebox[origin=c]{4}{x}} \right)$ are given functions the relations (8) and (15) give the following inequality;

16 $$\left|\Delta J\left(k\right)\right|\le {c}_{2}\left({\Vert \Delta k\Vert }_{{L}_{2}\left(0,l\right)}\right).$$

This inequality implies the continuity of the functional $J\left(k\right)$ with the norm of ${L}_{2}\left(0,l\right)$ on the set $K$. Besides, the functional $J\left(k\right)$ is bounded from below on $K$. Aditionally, ${L}_{2}\left(0,l\right)$ is a uniformly convex reflexive Banach space and the set $K$ is closed and bounded in ${L}_{2}\left(0,l\right)$. Then, it follows from the study^11^ that there exists a dense subset $G$ of the space ${L}_{2}\left(0,l\right)$ such that for any ${k}^{+}\in G$ the problem (7) with $\alpha >0$ has a unique ${k}^{+}$-minimum norm solution.

## Characterization of the solution

We introduce the following Lagrange functional for the problem (10);

17 $$\begin{aligned} L\left(\calligra{\rotatebox[origin=c]{4}{u}},k,\eta \right)= & \; {\beta }_{1}\underset{0}{\overset{l}{\int }} {\left[\calligra{\rotatebox[origin=c]{4}{u}} \left(\calligra{\rotatebox[origin=c]{4}{x}} ,T;k\right)-{y}_{1} \left(\calligra{\rotatebox[origin=c]{4}{x}} \right)\right]}^{2}d\calligra{\rotatebox[origin=c]{4}{x}}+{\beta }_{2}\underset{0}{\overset{l}{\int }}{\left[{\calligra{\rotatebox[origin=c]{4}{u}}}_{t} \left(\calligra{\rotatebox[origin=c]{4}{x}} ,T;k\right)-{y}_{2} \left(\calligra{\rotatebox[origin=c]{4}{x}} \right)\right]}^{2}d\calligra{\rotatebox[origin=c]{4}{x}} \\ \quad &+\alpha {\int }_{0}^{l}{\left[k-{k}^{+}\right]}^{2}d\calligra{\rotatebox[origin=c]{4}{x}}+\underset{0}{\overset{T}{\int }}\underset{0}{\overset{l}{\int }}\left({\rho \left(\calligra{\rotatebox[origin=c]{4}{x}} \right)\calligra{\rotatebox[origin=c]{4}{u}}}_{tt}-{\left(k \left(\calligra{\rotatebox[origin=c]{4}{x}} \right){\calligra{\rotatebox[origin=c]{4}{u}}}_{\calligra{\rotatebox[origin=c]{4}{x}}}\right)}_{\calligra{\rotatebox[origin=c]{4}{x}}}-f\right)\eta d\calligra{\rotatebox[origin=c]{4}{x}}dt. \end{aligned}$$

Let $\delta u$ be an increment for $u$ such that $\delta u \left(\calligra{\rotatebox[origin=c]{4}{x}} ,0\right)=\delta {u}_{t} \left(\calligra{\rotatebox[origin=c]{4}{x}} ,0\right)=\delta u\left(0,t\right)=\delta u\left(l,t\right)=0$ then the first variation of this functional is

$$\begin{aligned} \delta L\left(\calligra{\rotatebox[origin=c]{4}{u}},k,\eta \right)= & \; \underset{0}{\overset{l}{\int }}{2\beta }_{1}\left[\calligra{\rotatebox[origin=c]{4}{u}} \left(\calligra{\rotatebox[origin=c]{4}{x}} ,T;k\right)-{y}_{1} \left(\calligra{\rotatebox[origin=c]{4}{x}} \right)\right]\delta \calligra{\rotatebox[origin=c]{4}{u}} \left(\calligra{\rotatebox[origin=c]{4}{x}} ,T\right)d\calligra{\rotatebox[origin=c]{4}{x}}+\underset{0}{\overset{l}{\int }}{2\beta }_{2}\left[{\calligra{\rotatebox[origin=c]{4}{u}}}_{t} \left(\calligra{\rotatebox[origin=c]{4}{x}} ,T;k\right)-{y}_{2} \left(\calligra{\rotatebox[origin=c]{4}{x}} \right)\right]\delta {\calligra{\rotatebox[origin=c]{4}{u}}}_{t} \left(\calligra{\rotatebox[origin=c]{4}{x}} ,T\right)d\calligra{\rotatebox[origin=c]{4}{x}}\\ \quad & +\underset{0}{\overset{T}{\int }}\underset{0}{\overset{l}{\int }}\left(\rho \left(\calligra{\rotatebox[origin=c]{4}{x}} \right){\delta \calligra{\rotatebox[origin=c]{4}{u}}}_{tt}-{\left(k \left(\calligra{\rotatebox[origin=c]{4}{x}} \right){\delta \calligra{\rotatebox[origin=c]{4}{u}}}_{x}\right)}_{x}\right)\eta d\calligra{\rotatebox[origin=c]{4}{x}}dt. \end{aligned}$$

By parts of integration, we have

18 $$\begin{aligned}\delta L\left(\calligra{\rotatebox[origin=c]{4}{u}},k,\eta \right)= & \; \underset{0}{\overset{l}{\int }}\left\{{2\beta }_{1}\left[\calligra{\rotatebox[origin=c]{4}{u}} \left(\calligra{\rotatebox[origin=c]{4}{x}} ,T;k\right)-{y}_{1} \left(\calligra{\rotatebox[origin=c]{4}{x}} \right)\right]-p \left(\calligra{\rotatebox[origin=c]{4}{x}} \right){\eta }_{t} \left(\calligra{\rotatebox[origin=c]{4}{x}} ,T\right)\right\}\delta \calligra{\rotatebox[origin=c]{4}{u}} \left(\calligra{\rotatebox[origin=c]{4}{x}} ,T\right)d\calligra{\rotatebox[origin=c]{4}{x}} +\underset{0}{\overset{l}{\int }}\left\{{2\beta }_{2}\left[{\calligra{\rotatebox[origin=c]{4}{u}}}_{t} \left(\calligra{\rotatebox[origin=c]{4}{x}} ,T;k\right)-{y}_{2} \left(\calligra{\rotatebox[origin=c]{4}{x}} \right)\right]+\rho \left(\calligra{\rotatebox[origin=c]{4}{x}} \right)\eta \left(\calligra{\rotatebox[origin=c]{4}{x}} ,T\right)\right\}\delta {\calligra{\rotatebox[origin=c]{4}{u}}}_{t} \left(\calligra{\rotatebox[origin=c]{4}{x}} ,T\right)d\calligra{\rotatebox[origin=c]{4}{x}} \\ \quad &+\underset{0}{\overset{T}{\int }}\underset{0}{\overset{l}{\int }}\left(\rho \left(\calligra{\rotatebox[origin=c]{4}{x}} \right){\eta }_{tt}-{\left(k \left(\calligra{\rotatebox[origin=c]{4}{x}} \right){\eta }_{\calligra{\rotatebox[origin=c]{4}{x}}}\right)}_{\calligra{\rotatebox[origin=c]{4}{x}}}\right)\delta ud\calligra{\rotatebox[origin=c]{4}{x}}dt. \end{aligned}$$

The stationary condition $\delta L=0$ gives the following adjoint problem;

19 $$\rho \left(\calligra{\rotatebox[origin=c]{4}{x}} \right){\eta }_{tt}-{\left(k \left(\calligra{\rotatebox[origin=c]{4}{x}} \right){\eta }_{x}\right)}_{x}=0$$

20 $$\rho \left(\calligra{\rotatebox[origin=c]{4}{x}} \right){\eta \left(\calligra{\rotatebox[origin=c]{4}{x}} ,T\right)=-2\beta }_{2}\left[{\calligra{\rotatebox[origin=c]{4}{u}}}_{t} \left(\calligra{\rotatebox[origin=c]{4}{x}} ,T;k\right)-{y}_{2} \left(\calligra{\rotatebox[origin=c]{4}{x}} \right)\right]$$

21 $$\rho \left(\calligra{\rotatebox[origin=c]{4}{x}} \right){{\eta }_{t} \left(\calligra{\rotatebox[origin=c]{4}{x}} ,T\right)=2\beta }_{1}\left[\calligra{\rotatebox[origin=c]{4}{u}} \left(\calligra{\rotatebox[origin=c]{4}{x}} ,T;k\right)-{y}_{1} \left(\calligra{\rotatebox[origin=c]{4}{x}} \right)\right]$$

22 $$\eta \left(0,t\right)=0, \eta \left(l,t\right)=0$$

The adjoint problem (19)–(22) has unique $\eta \in {C}^{1}\left(\left[0,T\right],{L}_{2}\left(0,l\right)\right)\cap C\left(\left[0,T\right],{W}_{2}^{1}\left(0,l\right)\right)$ solution and this solution satisfies the following inequalities;

23 $$\begin{aligned} & \underset{0\le t\le T}{\mathrm{max}}{\left[{\Vert \eta \Vert }_{{L}_{2}\left(0,l\right)}^{2}+{\Vert {\eta }_{x}\Vert }_{{L}_{2}\left(0,l\right)}^{2}+{\Vert {\eta }_{t}\Vert }_{{L}_{2}\left(0,l\right)}^{2}\right]}^{1/2}\\ &{\le c}_{3}\left({\Vert f\Vert }_{{L}_{2}\left(\Omega \right)}+{\Vert \varphi \Vert }_{{W}_{2}^{1}\left(0,l\right)}+{\Vert \psi \Vert }_{{L}_{2}\left(0,l\right)}+{\Vert {y}_{1}\left(x\right)\Vert }_{{L}_{2}\left(0,l\right)}+{\Vert {y}_{2}\left(x\right)\Vert }_{{L}_{2}\left(0,l\right)}\right) \end{aligned}$$

or

24 $$\underset{0\le t\le T}{\mathrm{max}}{\left[{\Vert \eta \Vert }_{{L}_{2}\left(0,l\right)}^{2}+{\Vert {\eta }_{x}\Vert }_{{L}_{2}\left(0,l\right)}^{2}+{\Vert {\eta }_{t}\Vert }_{{L}_{2}\left(0,l\right)}^{2}\right]}^{1/2}\le {c}_{4}$$

where ${c}_{3}$ and ${c}_{4}$ are constants.

Now, we investigate the differentiability of the functional. By the definition of Fréchet differentiability, we can write

25 $$\Delta I\left(k\right)={\left\langle {I}^{\prime}\left(k\right),\Delta k \right\rangle }_{{L}_{2}\left(0,l\right)}+o\left({\Vert \Delta k\Vert }_{{L}_{2}\left(0,l\right)}\right)$$

with $\underset{\Vert \Delta k\Vert \to 0}{\mathrm{lim}}o\left(\Vert \Delta k\Vert \right)/\Vert \Delta k\Vert =0$ for any arbitrary $\Delta k\in {L}_{2}\left(0,l\right)$.

The difference of the functional according to the increment $\Delta k$ is such as

$$\Delta I\left(k\right)=\underset{0}{\overset{l}{\int }}{2\beta }_{1}\left[\calligra{\rotatebox[origin=c]{4}{u}} \left(\calligra{\rotatebox[origin=c]{4}{x}} ,T;k\right)-{y}_{1} \left(\calligra{\rotatebox[origin=c]{4}{x}} \right)\right]\Delta \calligra{\rotatebox[origin=c]{4}{u}} \left(\calligra{\rotatebox[origin=c]{4}{x}} ,T\right)d\calligra{\rotatebox[origin=c]{4}{x}}+\underset{0}{\overset{l}{\int }}{2\beta }_{2}\left[{\calligra{\rotatebox[origin=c]{4}{u}}}_{t} \left(\calligra{\rotatebox[origin=c]{4}{x}} ,T;k\right)-{y}_{2} \left(\calligra{\rotatebox[origin=c]{4}{x}} \right)\right]\Delta {\calligra{\rotatebox[origin=c]{4}{u}}}_{t} \left(\calligra{\rotatebox[origin=c]{4}{x}} ,T\right)d\calligra{\rotatebox[origin=c]{4}{x}}$$

26 $$+{\beta }_{1}\underset{0}{\overset{l}{\int }}{\left[\Delta \calligra{\rotatebox[origin=c]{4}{u}} \left(\calligra{\rotatebox[origin=c]{4}{x}} ,T\right)\right]}^{2}d\calligra{\rotatebox[origin=c]{4}{x}}+{\beta }_{2}\underset{0}{\overset{l}{\int }}{\left[\Delta {\calligra{\rotatebox[origin=c]{4}{u}}}_{t} \left(\calligra{\rotatebox[origin=c]{4}{x}} ,T\right)\right]}^{2}d\calligra{\rotatebox[origin=c]{4}{x}}+{\int }_{0}^{l}2\alpha \left(k-{k}^{+}\right)\Delta kd\calligra{\rotatebox[origin=c]{4}{x}}+\alpha {\int }_{0}^{l}{\left[\Delta k\right]}^{2}d\calligra{\rotatebox[origin=c]{4}{x}}$$

The first three integrals of the right side are to be evaluated by the solution of the adjoint problem. Hence, multiplying the Eq. (11) by the function $\eta$ and integrating over $\Omega$, we have

27 $$\underset{0}{\overset{T}{\int }}\underset{0}{\overset{l}{\int }}\left[p \left(\calligra{\rotatebox[origin=c]{4}{x}} \right){\Delta \calligra{\rotatebox[origin=c]{4}{u}}}_{tt}-{\left(k \left(\calligra{\rotatebox[origin=c]{4}{x}} \right){\Delta \calligra{\rotatebox[origin=c]{4}{u}}}_{\calligra{\rotatebox[origin=c]{4}{x}}}\right)}_{x}-{\left(\Delta k \left(\calligra{\rotatebox[origin=c]{4}{x}} \right){\Delta \calligra{\rotatebox[origin=c]{4}{u}}}_{x}\right)}_{x}-{\left(\Delta k \left(\calligra{\rotatebox[origin=c]{4}{x}} \right){\calligra{\rotatebox[origin=c]{4}{u}}}_{\calligra{\rotatebox[origin=c]{4}{x}}}\right)}_{x}\right]\eta d\calligra{\rotatebox[origin=c]{4}{x}}dt=0$$

By parts of integration and using adjoint problem, we get

$$\begin {aligned} \underset{0}{\overset{T}{\int }}\underset{0}{\overset{l}{\int }}p \left(\calligra{\rotatebox[origin=c]{4}{x}} \right){\Delta \calligra{\rotatebox[origin=c]{4}{u}}}_{tt}\eta dxdt= & \underset{0}{\overset{l}{\int }}{2\beta }_{1}\left[\calligra{\rotatebox[origin=c]{4}{u}} \left(\calligra{\rotatebox[origin=c]{4}{x}} ,T;k\right)-{y}_{1} \left(\calligra{\rotatebox[origin=c]{4}{x}} \right)\right]\Delta \calligra{\rotatebox[origin=c]{4}{u}} \left(\calligra{\rotatebox[origin=c]{4}{x}} ,T\right)d\calligra{\rotatebox[origin=c]{4}{x}} \\ \quad &+\underset{0}{\overset{l}{\int }}{2\beta }_{2}\left[{\calligra{\rotatebox[origin=c]{4}{u}}}_{t} \left(\calligra{\rotatebox[origin=c]{4}{x}} ,T;k\right)-{y}_{2} \left(\calligra{\rotatebox[origin=c]{4}{x}} \right)\right]\Delta {\calligra{\rotatebox[origin=c]{4}{u}}}_{t} \left(\calligra{\rotatebox[origin=c]{4}{x}} ,T\right)d\calligra{\rotatebox[origin=c]{4}{x}}+\underset{0}{\overset{T}{\int }}\underset{0}{\overset{l}{\int }}p \left(\calligra{\rotatebox[origin=c]{4}{x}} \right){\eta }_{tt}\Delta \calligra{\rotatebox[origin=c]{4}{u}}d\calligra{\rotatebox[origin=c]{4}{x}}dt \\ \underset{0}{\overset{T}{\int }}\underset{0}{\overset{l}{\int }}{\left(-k \left(\calligra{\rotatebox[origin=c]{4}{x}} \right){\Delta \calligra{\rotatebox[origin=c]{4}{u}}}_{x}\right)}_{x}\eta d\calligra{\rotatebox[origin=c]{4}{x}}dt & =\underset{0}{\overset{T}{\int }}\underset{0}{\overset{l}{\int }}{\left(-{k \left(\calligra{\rotatebox[origin=c]{4}{x}} \right)\eta }_{x}\right)}_{x}\Delta ud\calligra{\rotatebox[origin=c]{4}{x}}dt \\ \underset{0}{\overset{T}{\int }}\underset{0}{\overset{l}{\int }}-{\left(\Delta k \left(\calligra{\rotatebox[origin=c]{4}{x}} \right){\Delta \calligra{\rotatebox[origin=c]{4}{u}}}_{x}\right)}_{x}\eta d\calligra{\rotatebox[origin=c]{4}{x}}dt &=\underset{0}{\overset{T}{\int }}\underset{0}{\overset{l}{\int }}\Delta {k \left(\calligra{\rotatebox[origin=c]{4}{x}} \right){\Delta \calligra{\rotatebox[origin=c]{4}{u}}}_{\calligra{\rotatebox[origin=c]{4}{x}}}\eta }_{\calligra{\rotatebox[origin=c]{4}{x}}}d\calligra{\rotatebox[origin=c]{4}{x}}dt \end {aligned}$$

and

$$\underset{0}{\overset{T}{\int }}\underset{0}{\overset{l}{\int }}-{\left(\Delta k \left(\calligra{\rotatebox[origin=c]{4}{x}} \right){\calligra{\rotatebox[origin=c]{4}{u}}}_{x}\right)}_{x}\eta dxdt =\underset{0}{\overset{T}{\int }}\underset{0}{\overset{l}{\int }}\Delta {k \left(\calligra{\rotatebox[origin=c]{4}{x}} \right){\calligra{\rotatebox[origin=c]{4}{u}}}_{x}\eta }_{x}d\calligra{\rotatebox[origin=c]{4}{x}}dt.$$

Considering these equalities in (28), we write

$$\begin{aligned} & \underset{0}{\overset{l}{\int }}{2\beta }_{1}\left[\calligra{\rotatebox[origin=c]{4}{u}} \left(\calligra{\rotatebox[origin=c]{4}{x}} ,T;k\right)-{y}_{1} \left(\calligra{\rotatebox[origin=c]{4}{x}} \right)\right]\Delta \calligra{\rotatebox[origin=c]{4}{u}} \left(\calligra{\rotatebox[origin=c]{4}{x}} ,T\right)d\calligra{\rotatebox[origin=c]{4}{x}}+\underset{0}{\overset{l}{\int }}{2\beta }_{2}\left[{\calligra{\rotatebox[origin=c]{4}{u}}}_{t} \left(\calligra{\rotatebox[origin=c]{4}{x}} ,T;k\right)-{y}_{2} \left(\calligra{\rotatebox[origin=c]{4}{x}} \right)\right]\Delta {\calligra{\rotatebox[origin=c]{4}{u}}}_{t} \left(\calligra{\rotatebox[origin=c]{4}{x}} ,T\right)d\calligra{\rotatebox[origin=c]{4}{x}} \\ = & \;\underset{0}{\overset{T}{\int }}\underset{0}{\overset{l}{\int }}\Delta {k \left(\calligra{\rotatebox[origin=c]{4}{x}} \right){\Delta \calligra{\rotatebox[origin=c]{4}{u}}}_{\calligra{\rotatebox[origin=c]{4}{x}}}\eta }_{\calligra{\rotatebox[origin=c]{4}{x}}}d\calligra{\rotatebox[origin=c]{4}{x}}dt+\underset{0}{\overset{T}{\int }}\underset{0}{\overset{l}{\int }}\Delta {k \left(\calligra{\rotatebox[origin=c]{4}{x}} \right){\calligra{\rotatebox[origin=c]{4}{u}}}_{\calligra{\rotatebox[origin=c]{4}{x}}}\eta }_{\calligra{\rotatebox[origin=c]{4}{x}}}d\calligra{\rotatebox[origin=c]{4}{x}}dt \end{aligned}$$

So, we have

28 $$\begin{aligned} & \underset{0}{\overset{l}{\int }}{2\beta }_{1}\left[\calligra{\rotatebox[origin=c]{4}{u}} \left(\calligra{\rotatebox[origin=c]{4}{x}} ,T;k\right)-{y}_{1} \left(\calligra{\rotatebox[origin=c]{4}{x}} \right)\right]\Delta \calligra{\rotatebox[origin=c]{4}{u}} \left(\calligra{\rotatebox[origin=c]{4}{x}} ,T\right)d\calligra{\rotatebox[origin=c]{4}{x}}+\underset{0}{\overset{l}{\int }}{2\beta }_{2}\left[{\calligra{\rotatebox[origin=c]{4}{u}}}_{t} \left(\calligra{\rotatebox[origin=c]{4}{x}} ,T;k\right)-{y}_{2} \left(\calligra{\rotatebox[origin=c]{4}{x}} \right)\right]\Delta {\calligra{\rotatebox[origin=c]{4}{u}}}_{t} \left(\calligra{\rotatebox[origin=c]{4}{x}} ,T\right)d\calligra{\rotatebox[origin=c]{4}{x}} \\ = & \; \underset{0}{\overset{T}{\int }}\underset{0}{\overset{l}{\int }}\Delta {k \left(\calligra{\rotatebox[origin=c]{4}{x}} \right){\calligra{\rotatebox[origin=c]{4}{u}}}_{\calligra{\rotatebox[origin=c]{4}{x}}}\eta }_{\calligra{\rotatebox[origin=c]{4}{x}}}d\calligra{\rotatebox[origin=c]{4}{x}}dt+{R}_{1} \end{aligned}$$

where $${R}_{1}=\underset{0}{\overset{T}{\int }}\underset{0}{\overset{l}{\int }}\Delta {k \left(\calligra{\rotatebox[origin=c]{4}{x}} \right){\Delta \calligra{\rotatebox[origin=c]{4}{u}}}_{\calligra{\rotatebox[origin=c]{4}{x}}}\eta }_{\calligra{\rotatebox[origin=c]{4}{x}}}d\calligra{\rotatebox[origin=c]{4}{x}}dt$$.

We conclude from (15) and (24) that.

$${R}_{1}=o\left({\Vert \Delta k\Vert }_{{L}_{\infty }\left(0,l\right)}\right.)$$

On the other hand, if we assign in (26) that

$${R}_{2}={\beta }_{1}\underset{0}{\overset{l}{\int }}{\left[\Delta \calligra{\rotatebox[origin=c]{4}{u}} \left(\calligra{\rotatebox[origin=c]{4}{x}} ,T\right)\right]}^{2}d\calligra{\rotatebox[origin=c]{4}{x}}+{\beta }_{2}\underset{0}{\overset{l}{\int }}{\left[\Delta {\calligra{\rotatebox[origin=c]{4}{u}}}_{t} \left(\calligra{\rotatebox[origin=c]{4}{x}} ,T\right)\right]}^{2}d\calligra{\rotatebox[origin=c]{4}{x}}+\alpha {\int }_{0}^{l}{\left[\Delta k\right]}^{2}d\calligra{\rotatebox[origin=c]{4}{x}}$$

then by (16) we obtain $\left|{R}_{2}\right|\le {c}_{6}{\Vert \Delta k\Vert }_{{L}_{\infty }\left(0,l\right)}$ and the relation;

29 $${R}_{1}+{R}_{2}=o\left({\Vert \Delta k\Vert }_{{L}_{2}\left(0,l\right)}\right).$$

Taking into account (28) and (29) in (26), we write the followings;

30 $$\begin{aligned} \Delta I\left(k\right)=& \;\underset{0}{\overset{T}{\int }}\underset{0}{\overset{l}{\int }}{\calligra{\rotatebox[origin=c]{4}{u}}}_{\calligra{\rotatebox[origin=c]{4}{x}}}{\eta }_{\calligra{\rotatebox[origin=c]{4}{x}}}\Delta kd\calligra{\rotatebox[origin=c]{4}{x}}dt+{\int }_{0}^{l}2\alpha \left(k-{k}^{+}\right)\Delta kd\calligra{\rotatebox[origin=c]{4}{x}}+o\left({\Vert \Delta k\Vert }_{{L}_{2}\left(0,l\right)}\right) \\ \Delta I\left(k\right)= & \;{\left\langle \underset{0}{\overset{T}{\int }}{\calligra{\rotatebox[origin=c]{4}{u}}}_{\calligra{\rotatebox[origin=c]{4}{x}}}{\eta }_{\calligra{\rotatebox[origin=c]{4}{x}}}dt+2\alpha \left(k-{k}^{+}\right),\Delta k\right\rangle }_{{L}_{2}\left(0,l\right)}+o\left({\Vert \Delta k\Vert }_{{L}_{2}\left(0,l\right)}\right) \end{aligned}.$$

So, the Frechet differentiation (gradient) is obtained such as

31 $${I}^{\prime} \left(k\right)=\underset{0}{\overset{T}{\int }}{\calligra{\rotatebox[origin=c]{4}{u}}}_{\calligra{\rotatebox[origin=c]{4}{x}}}{\eta }_{\calligra{\rotatebox[origin=c]{4}{x}}}dt+2\alpha \left(k-{k}^{+}\right).$$

This gradient expression is used in the gradient descent iteration (32) to update the current estimate of the unknown coefficient at each step.

## Obtaining a minimizing sequence by gradient method

Upon selecting an initial element ${k}_{0}\left(x\right)\in K$, the gradient method necessitates of finding the sequence $\left\{{k}_{m}\right\}$ by

32 $$k_{m + 1} = k_{m} - \tau_{m} I^{\prime}\left( {k_{m} } \right),\tau_{m} > 0,m = 0,1,2, \ldots$$

Here, ${\tau }_{m}$ is algorithm parameter. If ${I}^{\prime}\left({k}_{m}\right)\ne 0$ then we select the quantity ${\tau }_{m}$ such that $I\left({k}_{m+1}\right)<I\left({k}_{m}\right)$. It can be directly obtained from (26) and (31) that for small enough ${\tau }_{m}>0$, we have

33 $$I\left( {k_{m + 1} } \right) - I\left( {k_{m} } \right) = \tau_{m} \left( { - I^{\prime}\left( {k_{m} } \right)^{2} + \frac{{o\left( {\tau_{m} } \right)}}{{\tau_{m} }}} \right) < 0.$$

If ${I}^{\prime}\left({k}_{m}\right)=0$ then ${k}_{m}$ is a stationary point of the problem (10). At this stage, the iteration of (32) is stopped and checked that the point ${k}_{m}$ is a solution of the problem (10) or not.

Given small enough $\varepsilon$, the iterations are stopped with the condition $\left|I\left({k}_{m}\right)-I\left({k}_{m+1}\right)\right|\le \varepsilon$. Until the condition $I\left({k}_{m+1}\right)<I\left({k}_{m}\right)$ holds for each value of $m$, the parameter ${\tau }_{m}>0$ must be decreased.

## Numerical illustrations

In this section we illustrate the theoretical results on two numerical examples and report sensitivity tests with respect to the regularization parameter α and the initial guess function . In addition, a noise-perturbation protocol is outlined to assess robustness when final-time observations are contaminated by measurement error.

Implementation details. All numerical simulations and graphical outputs were generated using MATLAB (MathWorks Inc.,USA). In our implementation we used MATLAB (MathWorks) and standard linear algebra routines.

Parameter choices. Unless otherwise stated, the gradient iteration (32) is started from the prior and terminated when the relative change criterion in (34) is met. The step size is selected by the backtracking strategy described above. The regularization weight α is chosen by testing a small set of candidate values and selecting a value that balances data fit and smoothness (in the spirit of the L-curve/discrepancy principle; cf.^4,5,10^).

### Example 1

Taking $\Omega =\left(\rm{0,1}\right)\times \left(\rm{0,1}\right)$, consider the issue;

$$\underset{k\in K}{\rm{min}}I\left(k\right)$$

where

$$I\left(k\right)=\underset{0}{\overset{l}{\int }}{\left|\calligra{\rotatebox[origin=c]{4}{u}} \left(\calligra{\rotatebox[origin=c]{4}{x}} ,1;k\right)-\calligra{\rotatebox[origin=c]{4}{x}} \left(\calligra{\rotatebox[origin=c]{4}{x}} -1\right)\right|}^{2}d\calligra{\rotatebox[origin=c]{4}{x}}+\underset{0}{\overset{l}{\int }}{\left|{\calligra{\rotatebox[origin=c]{4}{u}}}_{t} \left(\calligra{\rotatebox[origin=c]{4}{x}} ,1;k\right)-2\calligra{\rotatebox[origin=c]{4}{x}} \left(\calligra{\rotatebox[origin=c]{4}{x}} -1\right)\right|}^{2}d\calligra{\rotatebox[origin=c]{4}{x}}+\alpha {\Vert k-{k}^{+}\Vert }_{{L}_{2}\left(\rm{0,1}\right)}^{2}$$

for the wave equation;

$$3\frac{{\partial }^{2}u}{\partial {t}^{2}}-\frac{\partial }{\partial x}\left(2\frac{\partial u}{\partial x}\right)=6{\calligra{\rotatebox[origin=c]{4}{x}}}^{2}-4{t}^{2}-6\calligra{\rotatebox[origin=c]{4}{x}}, \left(\calligra{\rotatebox[origin=c]{4}{x}} ,t\right)\in\Omega$$

$$\calligra{\rotatebox[origin=c]{4}{u}} \left(\calligra{\rotatebox[origin=c]{4}{x}} ,0\right)=0, {\calligra{\rotatebox[origin=c]{4}{u}}}_{t} \left(\calligra{\rotatebox[origin=c]{4}{x}} ,0\right)=0, \calligra{\rotatebox[origin=c]{4}{x}}\in \left(\rm{0,1}\right)$$

$$\calligra{\rotatebox[origin=c]{4}{u}}\left(0,t\right)=\calligra{\rotatebox[origin=c]{4}{u}}\left(1,t\right)=0, t\in \left(\rm{0,1}\right)$$

In the cost functional, we will take ${k}^{+}=1$ and ${k}^{+}=2$, respectively and then investigate the $1$-minimum norm control and $2$-minimum norm control, using the minimizing sequence.

In the following table, there are some $\alpha$ values and corresponding minimum norm controls with $N=2$ sum. Here the starting element is ${k}_{0} \left(\calligra{\rotatebox[origin=c]{4}{x}} \right)=1$ and iteration stopping criteria is $I\left({k}_{m}\right)-I\left({k}_{m+1}\right)<0.01$ (Table 1).

These solutions produce the following functional and norm values;

**Table 1:** Some $\alpha$ values and corresponding ${k}_{*}(x)$ controls.

$\alpha$	${k}_{*}\left(x\right)$
0.1	$1+0.42176{ {\mathrm{cos}} }^{2}\pi x$ $\left({k}^{+}=1\right)$
	$1.46855+0.48325{ {\mathrm{cos}} }^{2}\pi x$ $\left({k}^{+}=2\right)$
0.2	$1+0.40865{ {\mathrm{cos}} }^{2}\pi x$ $\left({k}^{+}=1\right)$
	$1.76167+0.24943{ {\mathrm{cos}} }^{2}\pi x$ $\left({k}^{+}=2\right)$
0.3	$1+0.34054{ {\mathrm{cos}} }^{2}\pi x$ $\left({k}^{+}=1\right)$
0.4	$1+0.28378{ {\mathrm{cos}} }^{2}\pi x$ $\left({k}^{+}=1\right)$
0.5	$1+0.23649{ {\mathrm{cos}} }^{2}\pi x$ $\left({k}^{+}=1\right)$
	$1.99987+0.00622{ {\mathrm{cos}} }^{2}\pi x$ $\left({k}^{+}=2\right)$
0.6	$1+0.19707{ {\mathrm{cos}} }^{2}\pi x$ $\left({k}^{+}=1\right)$
0.8	$1+0.13685{ {\mathrm{cos}} }^{2}\pi x$ $\left({k}^{+}=1\right)$

**Fig. 1:**
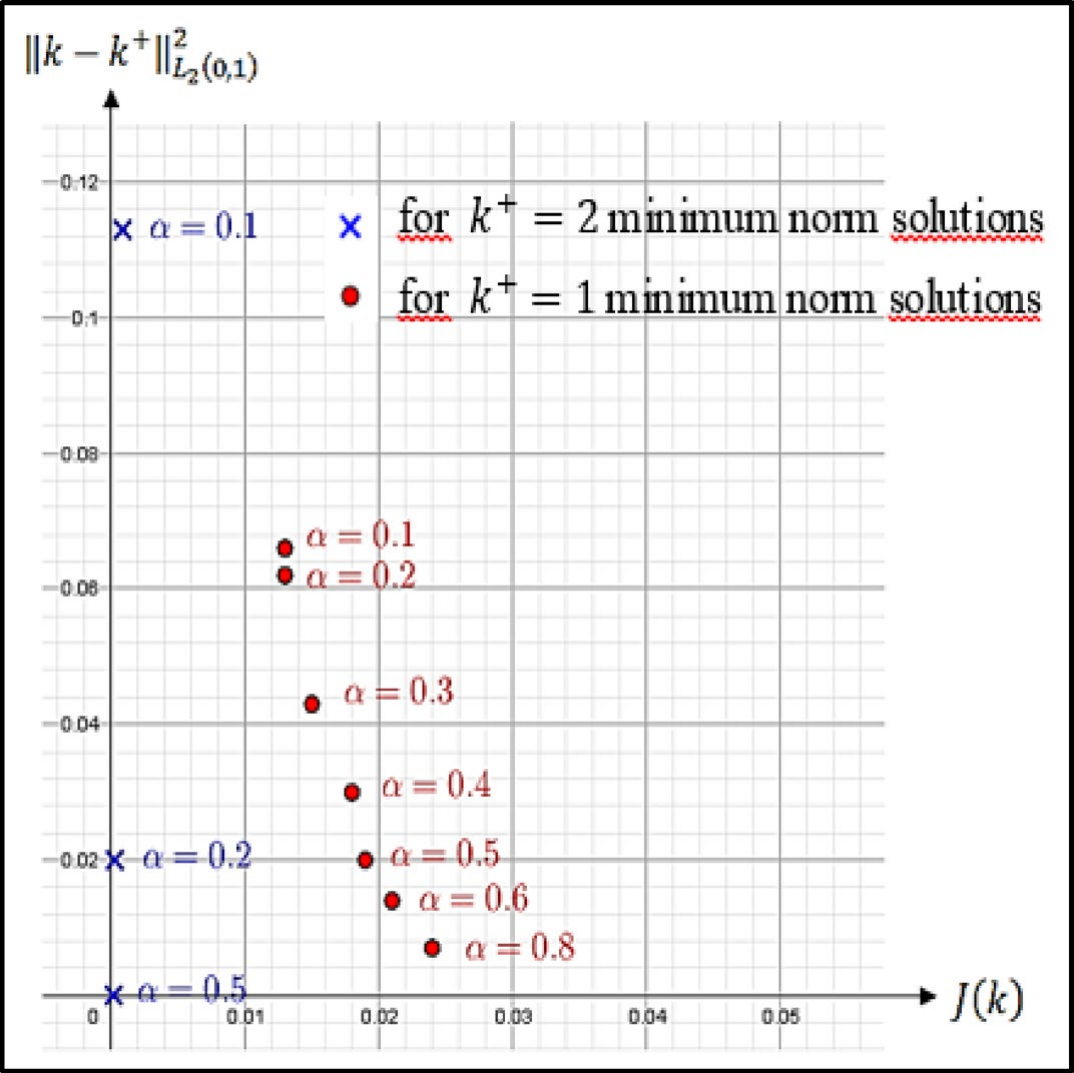
$J\left(k\right)$ and ${\Vert k-{k}^{+}\Vert }_{{L}_{2}\left(\mathrm{0,1}\right)}^{2}$ values for some $\alpha$ numbers.

**Fig. 2:**
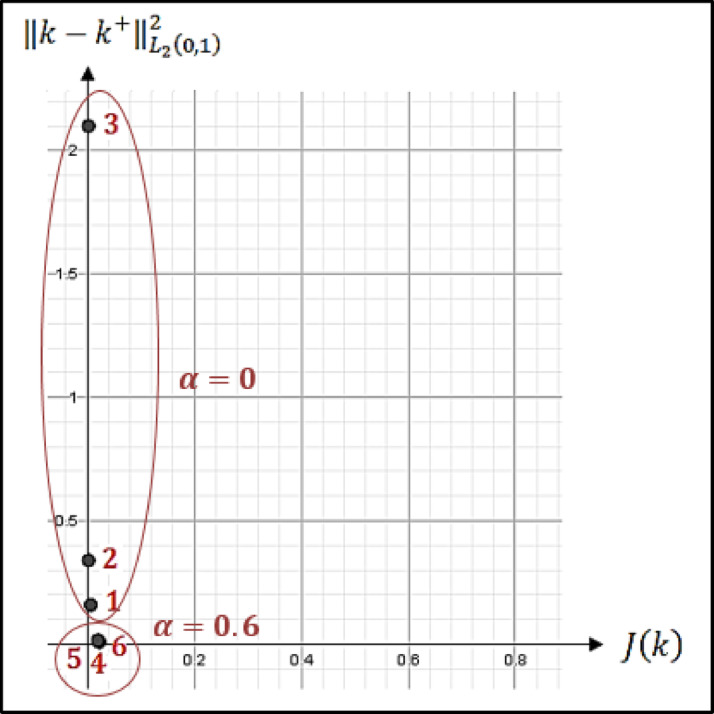
The effect of α parameter to the 1-minimum norm control with different starting elements.

**Fig. 3:**
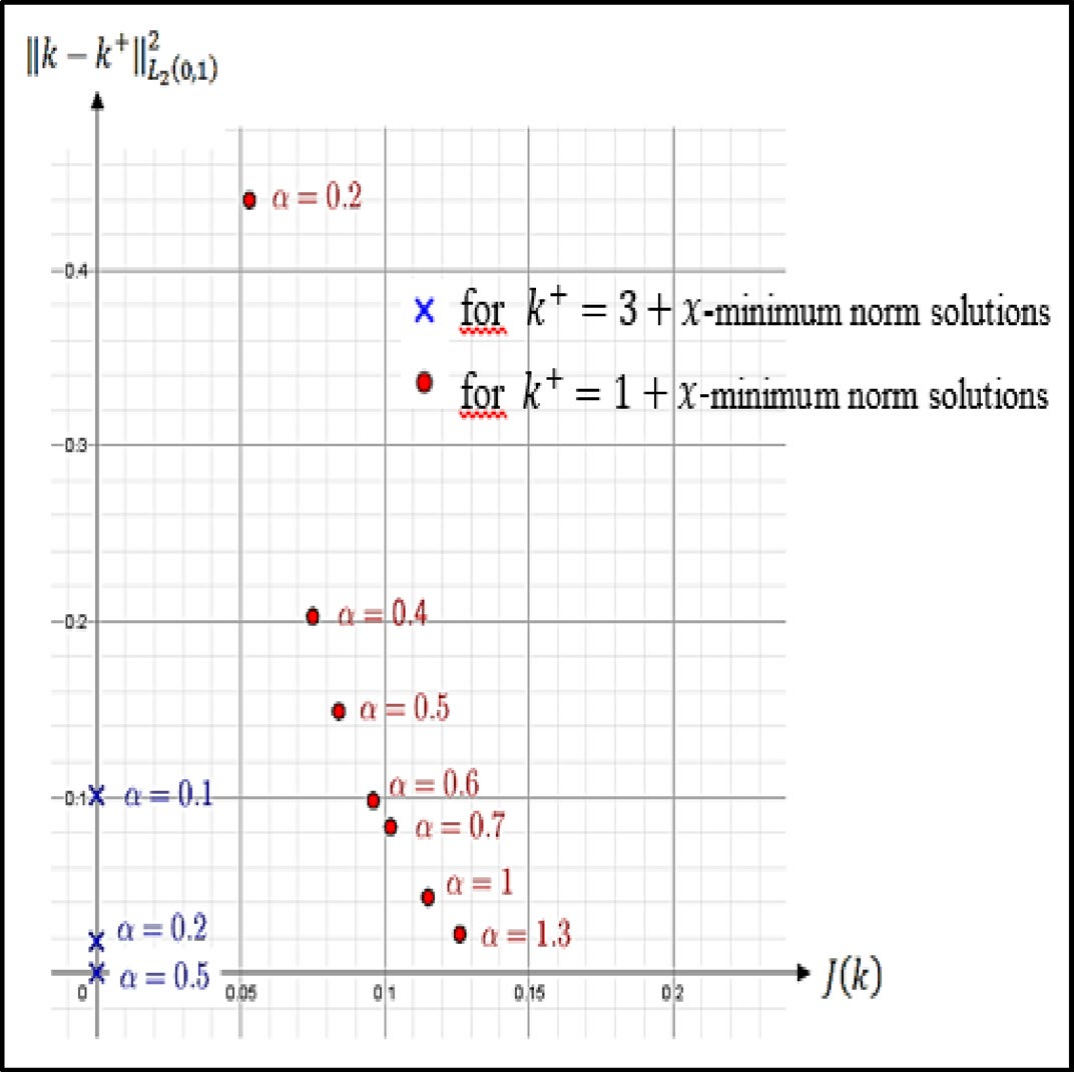
$J\left(k\right)$ and ${\Vert k-{k}^{+}\Vert }_{{L}_{2}\left(\mathrm{0,1}\right)}^{2}$ values for some $\alpha$ numbers.

**Fig. 4:**
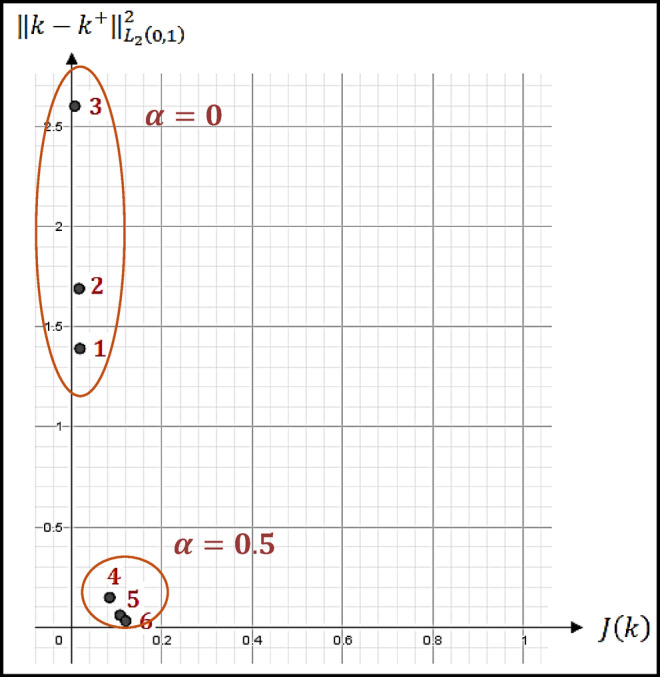
The influence of the parameters $\alpha =0$ and $\alpha =0.5$ to the solution for ${k}^{+}=1+x$.

**Table 2:** The ${k}^{+}=1$ and ${k}^{+}=2$ values in Fig. 1 are shown in the Table 2.

$\alpha$	${k}^{+}=1$		${k}^{+}=2$	
	$J\left(k\right)$	${\Vert k-{k}^{+}\Vert }_{{L}_{2}\left(\mathrm{0,1}\right)}^{2}$	$J\left(k\right)$	${\Vert k-{k}^{+}\Vert }_{{L}_{2}\left(\mathrm{0,1}\right)}^{2}$
$0.1$	$0.013$	$0.066$	$0.0009$	$0.113$
$0.2$	$0.013$	$0.062$	$0.0003$	$0.020$
$0.3$	$0.0159$	$0.043$		
$0.4$	$0.018$	$0.03$		
$0.5$	$0.0199$	$0.021$	$0.0002$	$0.00001$
$0.6$	$0.021$	$0.014$		
$0.8$	$0.024$	$0.007$		

**Table 3:** Some $\alpha$ and ${k}_{0}(x)$ values and corresponding $J\left(k\right)$ and $\left\| {k - k^{ + } } \right\|_{{L_{2} \left( {{\mathrm{0,1}}} \right)}}^{2}$ control.

Point	Starting element		$J\left(k\right)$	${\Vert k-{k}^{+}\Vert }_{{L}_{2}\left(\mathrm{0,1}\right)}^{2}$
1	${k}_{0}\left(x\right)=1$	for $\alpha =0$	$0.007$	$0.16$
2	${k}_{0}\left(x\right)=1.5$	for $\alpha =0$	$0.003$	$0.34$
3	${k}_{0}\left(x\right)=2.5$	for $\alpha =0$	$0.03$	$2.1$
4	${k}_{0}\left(x\right)=1$	for $\alpha =\mathrm{0,6}$	$0.021$	$0.014$
5	${k}_{0}\left(x\right)=1.5$	for $\alpha =\mathrm{0,6}$	$0.023$	$0.009$
6	${k}_{0}\left(x\right)=2.5$	for $\alpha =\mathrm{0,6}$	$0.02$	$0.016$

**Table 4:** Some $\alpha$ values and corresponding ${k}_{*}\left(x\right)$ controls.

$\alpha$	${k}_{*}\left(x\right)$
0.1	$1.810+0.910x+0.59431{ {\mathrm{cos}} }^{2}\pi x$ $\left({k}^{+}=1+x\right)$
	$2.65132+0.65132x+0.65900{ {\mathrm{cos}} }^{2}\pi x$ $\left({k}^{+}=3+x\right)$
0.2	$1.620+0.380x+0.59431{ {\mathrm{cos}} }^{2}\pi x$ $\left({k}^{+}=1+x\right)$
	$2.85223+0.85223x+0.28998{ {\mathrm{cos}} }^{2}\pi x$ $\left({k}^{+}=3+x\right)$
0.4	$1.05760+0.9424x+0.69696{ {\mathrm{cos}} }^{2}\pi x$ $\left({k}^{+}=1+x\right)$
0.5	$1.04232+0.95767x+0.60195{ {\mathrm{cos}} }^{2}\pi x$ $\left({k}^{+}=1+x\right)$ $2.99954+0.99954x-0.01249{ {\mathrm{cos}} }^{2}\pi x$ $\left({k}^{+}=3+x\right)$
0.6	$0.86863+1.13136x+0.593866{ {\mathrm{cos}} }^{2}\pi x$ $\left({k}^{+}=1+x\right)$
0.7	$0.84638+1.15361x+0.56214{ {\mathrm{cos}} }^{2}\pi x$ $\left({k}^{+}=1+x\right)$
1	$0.92759+1.07240x+0.38735{ {\mathrm{cos}} }^{2}\pi x$ $\left({k}^{+}=1+x\right)$
1.3	$0.91127+1.08872x+0.29710{ {\mathrm{cos}} }^{2}\pi x$ $\left({k}^{+}=1+x\right)$

**Table 5:** The ${k}^{+}=1+x$ and ${k}^{+}=3+x$ values in Fig. 1 are shown in the Table 2.

$\alpha$	${k}^{+}=1+x$		${k}^{+}=3+x$	
	$J\left(k\right)$	${\Vert k-{k}^{+}\Vert }_{{L}_{2}\left(\mathrm{0,1}\right)}^{2}$	$J\left(k\right)$	${\Vert k-{k}^{+}\Vert }_{{L}_{2}\left(\mathrm{0,1}\right)}^{2}$
$0.1$	$0.045$	$0.59$	$0$	$0.101$
$0.2$	$0.053$	$0.44$	$0$	$0.018$
$0.4$	$0.075$	$0.203$		
$0.5$	$0.084$	$0.149$	$0$	$0.00006$
$0.6$	$0.096$	$0.098$		
$0.7$	$0.102$	$0.083$		
$1$	$0.115$	$0.043$		
$1.3$	$0.126$	$0.022$		

**Table 6:** Some $\alpha$ and ${k}_{0}\left(x\right)$ values and corresponding $J\left(k\right)$ and ${\Vert k-{k}^{+}\Vert }_{{L}_{2}\left(\mathrm{0,1}\right)}^{2}$ control.

Point	Starting element		$J\left(k\right)$	${\Vert k-{k}^{+}\Vert }_{{L}_{2}\left(\mathrm{0,1}\right)}^{2}$
1	${k}_{0}\left(x\right)=2$	for $\alpha =0$	$0.018$	$1.39$
2	${k}_{0}\left(x\right)=2.5$	for $\alpha =0$	$0.016$	$1.69$
3	${k}_{0}\left(x\right)=3$	for $\alpha =0$	$0.007$	$2.6$
4	${k}_{0}\left(x\right)=2$	for $\alpha =\mathrm{0,5}$	$0.084$	$0.149$
5	${k}_{0}\left(x\right)=2.5$	for $\alpha =\mathrm{0,5}$	$0.107$	$0.061$
6	${k}_{0}\left(x\right)=3$	for $\alpha =\mathrm{0,5}$	$0.12$	$0.032$

It can be seen from these that the $2$-minimum norm controls arise more proper approximations than $1$-minimum norm controls. In order to investigate the effect of $\alpha$ parameter to the solution let us consider the $1$-minimum norm controls when $\alpha =0$ and $\alpha =0.6$ with different starting elements (Table 2).

Figure shows the influence of the starting element on the reconstruction. When $\alpha =0$, the obtained controls can vary significantly with different ${k}_{0}\left(x\right)$ initial guesses, whereas for $\alpha$>0 the reconstructed controls become less sensitive to initialization and their norms are closer across different ${k}_{0}\left(x\right)$ starting elements (Fig. 2, Table 3).

### Example 2

Taking $\Omega =\left(\rm{0,1}\right)\times \left(\rm{0,1}\right)$, consider the issue;

$$\underset{k\in K}{\rm{min}}I\left(k\right)$$

where

$$I\left(k\right)=\underset{0}{\overset{1}{\int }}{\left|\calligra{\rotatebox[origin=c]{4}{u}}\left(x,1;k\right)- {\rm{sin}} \pi x\right|}^{2}dx+\underset{0}{\overset{1}{\int }}{\left|{\calligra{\rotatebox[origin=c]{4}{u}}}_{t}\left(x,1;k\right)-0\right|}^{2}dx+\alpha {\Vert k-{k}^{+}\Vert }_{{L}_{2}\left(\rm{0,1}\right)}^{2}$$

for the wave equation;

$$\left( {2 + x} \right)\frac{{\partial^{2} u}}{{\partial t^{2} }} - \frac{\partial }{\partial x}\left( {\left( {3 + x} \right)\frac{\partial u}{{\partial x}}} \right) = - \pi \cos 2\pi t\left[ {\left( {3\pi x + 5\pi } \right)\sin \pi x + \cos \pi x} \right],{ }\left( {x,t} \right) \in {\Omega }$$

$$\calligra{\rotatebox[origin=c]{4}{u}}\left(x,0\right)= {\rm{sin}} \pi x, {\calligra{\rotatebox[origin=c]{4}{u}}}_{t}\left(x,0\right)=0, x\in \left(\rm{0,1}\right)$$

$$\calligra{\rotatebox[origin=c]{4}{u}}\left(0,t\right)=\calligra{\rotatebox[origin=c]{4}{u}}\left(1,t\right)=0, t\in \left(\rm{0,1}\right)$$

In the cost functional, we will take ${k}^{+}=1+x$ and ${k}^{+}=3+x$, respectively and then investigate the $1+x$-minimum norm control and $3+x$-minimum norm control, using the minimizing sequence. In the following table, there are some $\alpha$ values and corresponding minimum norm controls with $N=2$ sum. Here the starting element is ${k}_{0}\left(x\right)=2$ and iteration stopping criteria is $I\left({k}_{m}\right)-I\left({k}_{m+1}\right)<0.01$ (Table 4).

These solutions give the following functional and norm values (Fig. 3, Table 5);

The computed values indicate that the $3+x$ regularized (α-minimum norm) controls provide a more stable compromise between data fit and control magnitude than the unregularized case.

Now, we will investigate the effect of $\alpha$ parameter to the solution. To do this, let us investigate $x$-minimum norm controls when $\alpha =0$ and $\alpha =0.5$ with different starting elements.

The figure also illustrates sensitivity with respect to the ${k}_{0} \left(\calligra{\rotatebox[origin=c]{4}{x}} \right)$ starting element. In the unregularized setting $\alpha =0$, the obtained controls may vary noticeably with different initial guesses. Introducing regularization $\alpha >0$ reduces this sensitivity and leads to reconstructions with closer norms across different ${k}_{0} \left(\calligra{\rotatebox[origin=c]{4}{x}} \right)$ starting elements (Fig. 4, Table 6).

## Noise-perturbation test (robustness)

In practical applications, the final-time observations are contaminated by measurement noise. To evaluate robustness, we perturb the synthetic final data by adding relative noise of level δ, e.g., and (with independent standard normal perturbations). For several noise levels (e.g., δ ∈ {1%, 5%, 10%}), we solve the regularized problem (10) and monitor the reconstruction error and the residual. The regularization weight α can be selected by the discrepancy principle (matching the residual to the noise level) or by generalized cross-validation; see^4,5,10^. Similar noisy-data protocols are standard in imaging inverse problems such as bioluminescence tomography and photoacoustic tomography^12–14^.

## Result and discussion

$J\left({k}^{+} \right)=\underset{k\in K}{\mathrm{min}}J\left(k\right)$ Examples 1–2 indicate that the regularization parameter α is the primary stabilizing factor. In the unregularized case $(\alpha =0)$ , the minimization may admit multiple minimizers and the recovered control/coefficient can depend on the starting element. Introducing $\alpha >0$ yields a well-posed regularized problem and reduces sensitivity to initialization, in line with general regularization theory^4,5^.

$\alpha$ Role of the prior/initial guess. The function ${k}^{+}$ plays a dual role: it serves as a prior estimate in the Tikhonov term $\alpha {\Vert k-{k}^{+}\Vert }_{{L}_{2}\left(0,l\right)}^{2}$ and it provides the starting point of the gradient iteration. When preliminary information is available, selecting ${k}^{+}$ accordingly can improve convergence and guide the reconstruction toward physically plausible coefficients. When preliminary information about the coefficient is available, choosing ${k}^{+}$ accordingly can improve convergence and guide the reconstruction toward physically plausible solutions. In the absence of prior knowledge, a smooth baseline function may be used. The parameter $\alpha$ can then be selected using criteria that balance data misfit and regularity, such as the L-curve method, the discrepancy principle, or generalized cross-validation; see^4,5,10^

For noisy final-time data, larger values of $\alpha$ help suppress noise amplification. The noise-perturbation protocol described in Sect. 6 provides a practical framework for assessing the stability of the reconstruction.

Compared with alternative regularization strategies such as truncated SVD or iterative regularization and GCV-based parameter selection^10^, or sparsity-promoting penalties frequently used in inverse imaging problems like bioluminescence tomography and photoacoustic tomography^12–14^ the quadratic Tikhonov regularization term adopted here is particularly suitable when the unknown tension coefficient is expected to be smooth.

Inverse problems involving discontinuous or nonlinear coefficients present additional challenges. For hyperbolic equations with discontinuous coefficients, see Hurd and Sattinger^15^. Carleman-based recovery in transmission settings is studied in Baudouin et al.^16^. Nonlinear and quasilinear wave inverse problems are analyzed in Romanov^17^. These works motivate future extensions of the present framework to more complex coefficient structures.

## Conclusion

This work studied an inverse coefficient (tension) identification problem for the wave equation and stabilized it using a quadratic Tikhonov regularization with a prior coefficient. Existence and uniqueness were established for the regularized problem, and an adjoint-based Fréchet gradient was derived, leading to an efficient gradient descent reconstruction algorithm. The hybrid Galerkin–Laplace numerical implementation and the reported numerical experiments demonstrate the important roles of the regularization parameter $\alpha$ and the prior ${k}^{+}$ in ensuring stability and reducing sensitivity to initialization. Future research directions include the automatic selection of $\alpha$ via discrepancy principles, L-curve, or generalized cross-validation criteria; robust extensions to discontinuous or nonlinear coefficients (cf.^15–17^); higher-dimensional formulations supported by efficient solvers; and systematic comparisons with alternative regularization approaches, including sparsity-promoting penalties widely used in inverse imaging. Potential application areas of the proposed framework include optical molecular imaging, bioluminescence tomography, and photoacoustic tomography, where regularization plays a central role in achieving stable reconstructions^18–21^.

## Data Availability

The datasets generated and/or analysed during the current study are available from the corresponding author upon reasonable request.

## Code availability

The MATLAB codes used to perform the numerical simulations and generate the results in this study are available from the corresponding author upon reasonable request.
